# Comparative evaluation of four fully automated immunoanalyzers for the simultaneous detection of HBeAg and Anti-HBe

**DOI:** 10.1371/journal.pone.0331381

**Published:** 2025-09-03

**Authors:** Junhyup Song, Seung Jun Choi, Sinyoung Kim, Younhee Park

**Affiliations:** Departments of Laboratory Medicine, Severance Hospital, Yonsei University College of Medicine, Seoul, Republic of Korea; St Paul's Hospital Millennium Medical College, ETHIOPIA

## Abstract

**Background:**

Hepatitis B envelope Antigen (HBeAg) and anti-hepatitis B envelope Antigen (anti-HBe) are crucial markers for evaluating hepatitis B virus infection status and guiding clinical decisions. Considering the increasing prevalence of HBeAg-negative variants, accurate detection of both markers is essential. This study aimed to examine the analytical performance of four fully automated immunoanalyzers for the simultaneous detection of HBeAg and Anti-HBe and to assess the inter-platform concordance.

**Methods:**

In total, 439 leftover serum samples from routine clinical testing for HBeAg or anti-HBe testing were analyzed using four immunoanalyzers: Architect i2000 and Alinity i (Abbott), Cobas e801 (Roche), and Atellica IM 1600 (Siemens). Samples not tested immediately were stored at 4 °C and analyzed within 24 hours of storage. Verification of Precision followed the CLSI EP15-A3 guidelines, and methods were compared according to the CLSI EP09-A3 guidelines. Qualitative agreement was assessed using Cohen’s κ, whereas quantitative correlation was evaluated using Deming regression and R² values.

**Results:**

Architect i2000 and Alinity i exhibited the highest correlation (R² = 0.9960) and concordance (κ = 0.953 for HBeAg, κ = 0.971 for anti-HBe). Cobas e801 showed strong overall correlation with Architect i2000 (R² = 0.9838), but displayed a systematic negative bias near the cutoff, substantially reducing qualitative agreement. Atellica IM 1600 demonstrated relatively weaker correlation with the other three platforms.

**Conclusions:**

Inter-platform discrepancies observed may impact clinical interpretation. As these discrepancies primarily stem from systematic bias, they can be readily mitigated by adjusting the reference values. Overall, further harmonization efforts are essential to enhance inter-platform agreement and ensure consistency in clinical decision-making.

## Introduction

Hepatitis B virus (HBV), first identified in 1966, is currently estimated to affect approximately 350 million individuals worldwide, with over 1 million deaths annually attributed to HBV-related chronic liver disease [1]. Although HBV infection can also manifest as acute hepatitis, the incidence of fulminant hepatitis among acute cases is < 1% [2]. In contrast, 15–40% of untreated patients with chronic hepatitis B (CHB) progress to cirrhosis and eventually face potentially fatal outcomes, namely complications such as decompensated cirrhosis or hepatocellular carcinoma [3–6]. Owing to its high mortality and long-term complications, chronic hepatitis B carries great significance as a global health burden. Implementing an accurate diagnostic approach for these patients is crucial to enable timely and appropriate curative interventions.

Initially, laboratory testing for chronic HBV infection heavily relied on the surface antigen, first discovered as the Australia antigen in the 1960s by Blumberg et al. [7]. Despite subsequent identification of the HBV core antigen (HBcAg), laboratory assays continued to target HBsAg in addition to anti-HBc antibodies owing to the absence of HBcAg in the peripheral blood of patients with CHB. Subsequently, another antigen, Hepatitis B envelope Antigen (HBeAg), was discovered to be circulating in the serum of patients with CHB [8]. Importantly, HBsAg remains positive in the majority of patients undergoing nucleos(t)ide analogue (NA) treatment. This is because HBsAg continues to be transcribed from covalently closed circular DNA, even when the reverse transcription of pregenomic RNA is effectively suppressed by NA [9]. In contrast, HBeAg usually becomes negative when immune clearance surpasses viral replication, making it a more suitable marker for viral replication [10,11].

Clinical diagnostic practices have evolved further with a deeper understanding of HBV and its clinical course. The discovery of HBeAg-negative mutants, which are increasingly prevalent in patients with chronic hepatitis B, has led to the recognition of the immune-escape or reactivation phase in the clinical course of CHB and highlighted the importance of determining the precise HBeAg status [12,13]. These mutants lack tolerogenic HBeAg and are associated with fulminant hepatitis and severe hepatic damage, despite their relatively low circulating viral load [14–16]. Therefore, continuous surveillance is required for patients in the immune-inactive phase, to better identify those with reactivated HBeAg-negative chronic hepatitis.

Consequently, the precise detection of HBeAg and/or anti-hepatitis B envelope Antigen (anti-HBe), along with other laboratory markers, including HBV DNA load, is essential for accurate disease monitoring and timely decision-making [17]. Various efforts have been made to comparatively evaluate immunoassays for HBeAg and anti-HBe and to optimize their clinical accuracy [18–21]. However, a direct comparison among the latest platforms, including a detailed performance analysis, has been lacking. Here, we assessed the analytical performance of four immunoassays from three different manufacturers and evaluated the concordance rate between their results.

## Materials and methods

### Study overview

The goal of this study is to evaluate and compare the analytical performance of four commercially available, fully automated HBeAg/Anti-HBe assay systems and assess the concordance rate among their results. First, we evaluated assay precision according to the clinical laboratory standard institute (CLSI) guideline EP15-A3 [22]. Second, we compared the assays according to the CLSI guideline EP09-A3 [23]. A total of 439 clinical samples, exceeding the minimum sample size of 100 recommended by the CLSI guidelines, were obtained and sequentially analyzed using the four systems.

### Ethical approval

This study was reviewed and approved by the institutional review board of Severance Hospital in Seoul, Korea (IRB No. 4-2024-0304). The need for informed consent was waived considering the nature of the study as a method comparison of laboratory instruments, on the condition that patient privacy was thoroughly protected and study involved minimal risk to patients.

### Automated immunoanalyzers and assay characteristics

Initially, the samples were measured using our existing equipment, the Architect i2000 (Abbott Diagnostics, Abbott Park, IL, USA), and subsequently, using three other high-throughput fully automated immunoanalyzers: the Alinity i (Abbott Diagnostics), Roche Cobas e801 (Roche Diagnostics GmbH, Mannheim, Germany), and Siemens Atellica IM 1600 (Siemens Healthineers, Tarrytown, NY, USA). The assays utilized in these systems were Alinity i HBeAg/Anti-HBe Reagent Kit, Architect HBeAg/Anti-HBe Reagent Kit, Elecsys HBeAg/Anti-HBe, and Atellica IM Hepatitis B e Antigen/Anti-Hepatitis B e Antigen, respectively. The same lot numbers of reagents and controls were used throughout the evaluation for all four assays/platforms.

The assay principles and specifications were obtained from the official product manuals and package inserts provided by the respective manufacturers. This information is summarized in Table 1. Each assay, except the Elecsys assay on Cobas e801, employs additional strategies for handling gray-zone results. The Architect and Alinity assays recommend additional confirmatory retesting before reporting the final reactive results. In the Atellica assay, a proactive gray-zone criterion is established, requiring a retest when the initial result falls within the gray zone. The final result is then determined based on the retest outcome.

**Table 1:** Characteristics of the Four Automated Immunoassay Systems Assays.

Principle	Architect i2000	Alinity i	Cobas e801	Atellica IM 1600
	CLIA	CLIA	ECLIA	CLIA
HBeAg	Sandwich	Sandwich	Sandwich	Sandwich
Anti-HBe	Competitive	Competitive	Competitive	Competitive
Tracers	Acridinium ester	Acridinium ester	Ruthenium complex	Acridinium ester
Minimum sample volume				
HBeAg	80 μL	80 μL	21 μL	100 μL
Anti-HBe	150 μL	150 μL	21 μL	100 μL
Interpretation				
HBeAg	S/CO < 1.0 Non-reactive	S/CO < 1.0 Non-reactive	COI < 1.0 Non-reactive	<0.80 Non-reactive
				0.800 Non-reactiveitest^b^
	S/CO Non-reactivetest^a^	S/CO Non-reactivetest^a^	COI Non-reactiv	≥OI Non-react
Anti-HBe	S/CO > 1.0 Non-reactive	S/CO > 1.0 Non-reactive	COI > 1.0 Non-reactive	<0.80 Non-reactive
				0.800 Non-Equivocal^c^
	S/CO ocal-reactivetest^a^	S/CO ocal-reactivetest^a^	COI ocal-reactiv	≥OI ocal-reac
Assay result (N = 419^d^)				
HBeAg				
Positives (%)	110 (26.3)	103 (24.6)	88 (21.0)	118 (28.2)
Negatives (%)	309 (73.7)	316 (75.4)	331 (79.0)	301 (71.8)
Anti-HBe				
Positives (%)	259 (61.8)	263 (62.8)	303 (72.3)	281 (67.1)
Negatives (%)	160 (38.2)	156 (37.2)	116 (27.7)	138 (32.9)

CLIA, chemiluminescence immunoassay; COI, cutoff index; ECLIA, electrochemiluminescence immunoassay; S/CO, signal to cut-off.

^a^Retest in duplicate. If both results are negative, the final result is negative. If either result is positive, the final result is positive.

^b^Retest in duplicate. If at least two of three results are < 1.00, the final result is negative, if at least two out of three results are ≥ 1.00, the final result is positive.

^c^Retest in duplicate. If the equivocal result persists after retesting, resampling, and retesting are recommended.

^d^Samples with anti-HBe values between 0.80 and 1.20 on the Atellica IM 1600 were excluded, as resampling and retesting were recommended for these cases.

### Inter-assay precision

Inter-assay precision was determined by measuring the control materials in four replicates over 5 d [22]. Precision values for each assay were evaluated using the respective control materials provided by the manufacturers.

### Sample collection and testing workflow

This study was conducted at Severance Hospital, a tertiary teaching hospital in South Korea. All samples submitted to our clinical laboratory for HBeAg or anti-HBe testing for any clinical purpose between May and August 2024 were included. The purpose of testing included confirmation of HBV infection when other markers are inconclusive, assessment of HBeAg positivity at initial diagnosis, and monitoring HBeAg seroconversion during treatment. Samples exhibiting significant hemolysis, hyperbilirubinemia, or lipemia were excluded. After routine testing using the Architect i2000, 439 leftover serum samples were secured and analyzed using the other three systems within 24 hours. Samples were stored at 4 °C if they were not analyzed immediately after routine testing.

### Statistical analyses

For comparing the qualitative results, samples with values between 0.80 and 1.20 on the Atellica IM 1600 were excluded from the analysis, as resampling and retesting were recommended for these cases. Qualitative agreement between two assay methods was assessed using Cohen’s κ, which provide a more robust comparison than simple percent agreement by accounting for agreement occurring by chance [24]. Relationships between quantitative values were analyzed using Deming regression, while correlations were assessed using the coefficient of determination (R^2^). Statistical analyses were performed using Graphpad Prism version 9 (GraphPad Software, La Jolla, CA, USA) and Analyze-it for Microsoft Excel 5.40 (Analyse-it Software Ltd, Leeds, UK). *P*-values <0.05 were considered statistically significant.

## Results

### Verification of assay precision

The precision of the three systems, excluding the i2000, was evaluated to verify the metrics suggested by the manufacturers. The results of the precision study are summarized in Table 2. For HBeAg, the coefficients of variation ranged from 4.53–65.9% for the negative control materials and 1.34–2.18% for the positive control materials. For anti-HBs, the coefficients of variation ranged from 1.56–31.87% for negative control materials and 2.15–3.35% for positive control materials.

**Table 2:** Precision of the Alinity I, Cobas e801, and Atellica IM systems.

Level	Aliniti i		Cobas e801		Atellica IM 1600	
	Negative	Positive	Negative	Positive	Negative	Positive
HBeAg						
Mean	0.27	4.04	0.11	14.14	0.07	7.47
SD	0.02	0.08	0.00	0.19	0.05	0.16
CV, %	7.85	2.07	4.53	1.32	65.9	2.18
Anti-HBe						
Mean	2.04	0.50	1.44	0.58	0.05	2.23
SD	0.05	0.01	0.02	0.01	0.01	0.07
CV, %	2.31	2.34	1.56	2.15	31.87	3.35

CV, coefficient of variation; SD, standard deviation.

### Comparison of assay index values among the four systems

**Fig 1:**
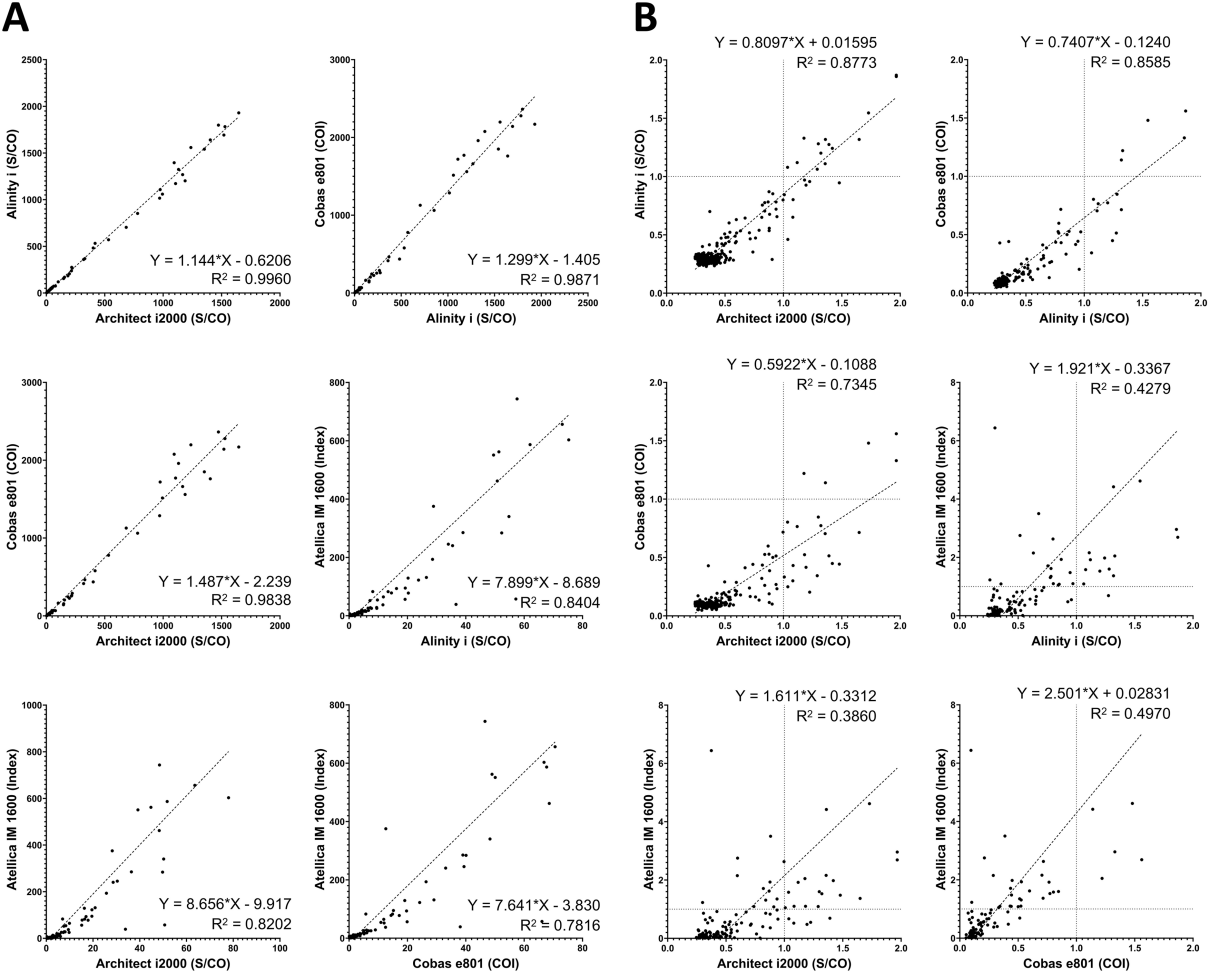
Pairwise comparison of HBeAg assay results obtained from four immunoanalyzers: Architect i2000, Alinity i, Cobas e801, and Atellica IM 1600. (A) Results from all 439 samples. (B) Results from 344 samples with values near the cutoff: below 2.0 for Architect i2000, Alinity i, and Cobas e801, and below 10.0 for Atellica IM 1600. Cutoff values for each analyzer are indicated by vertical or horizontal dotted lines.

Pairwise comparisons of assay index values for a total of 439 HBeAg results are presented in Fig 1A. The Architect i2000 and Alinity i showed the strongest correlation, with an R² value of 0.9960. The Cobas e801 also exhibited a high correlation with both the Architect i2000 and Alinity i, with R² values of 0.9838 and 0.9871, respectively. However, the Atellica IM 1600 showed a relatively lower correlation with the other three systems.

To better assess agreement in critical regions that may influence result interpretation, a subset of 344 results with values near the cutoff is presented separately in Fig 1B. The Architect i2000 and Alinity i showed the strongest correlation, whereas the Cobas e801 exhibited a systematic negative bias relative to the Architect i2000, with a slope of 0.5922. However, the negative bias of the Cobas e801 was less pronounced when compared to that of the Alinity i. The Atellica IM 1600, compared with the other three systems, exhibited generally lower agreement and a significant positive bias.

**Fig 2:**
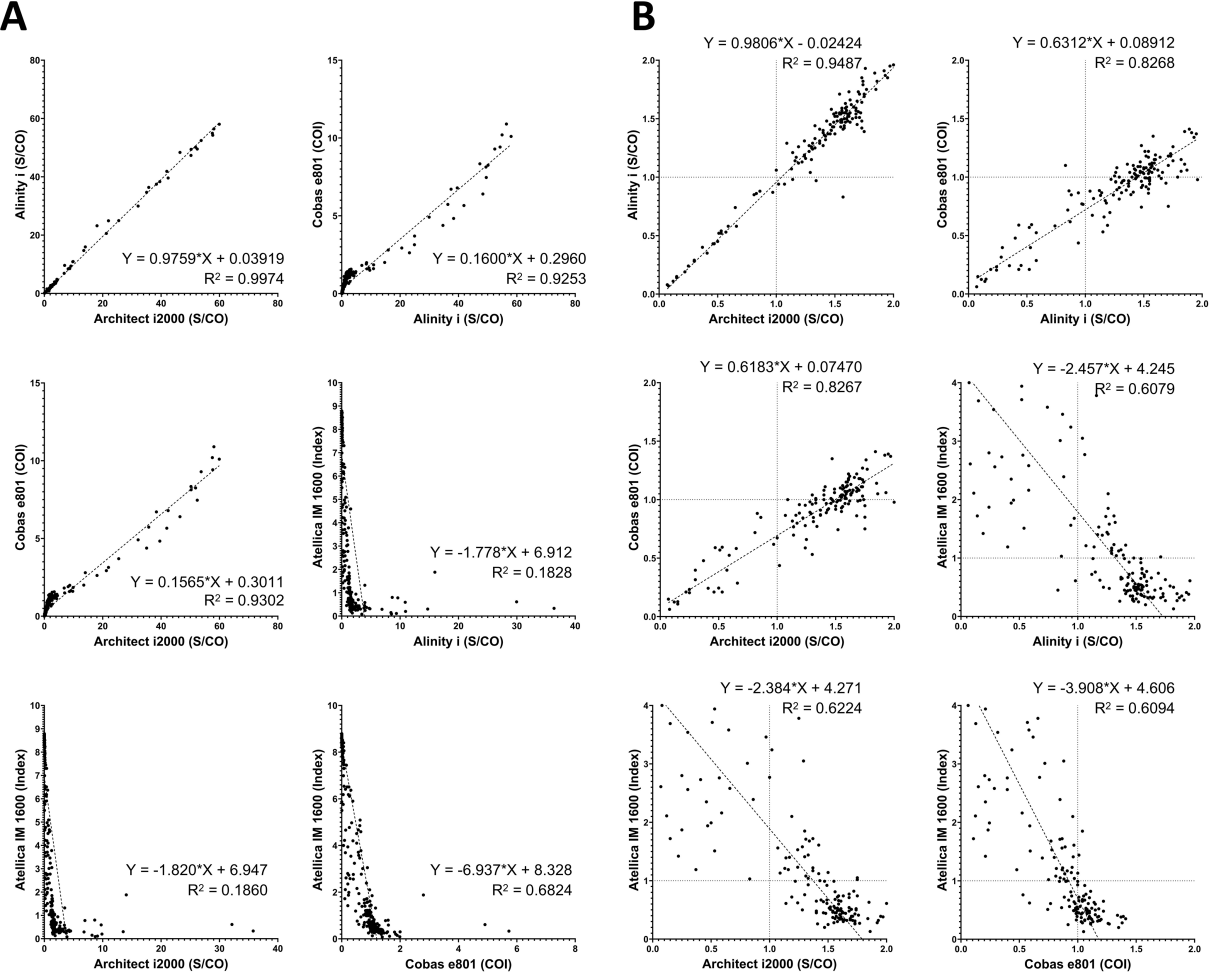
Pairwise comparison of the Anti-HBe assay results obtained from four immunoanalyzers: Architect i2000, Alinity i, Cobas e801, and Atellica IM 1600. (A) Results from all 439 samples. (B) Results from 146 samples with values near the cutoff: below 2.0 for Architect i2000, Alinity i, and Cobas e801, and below 4.0 for Atellica IM 1600. Cutoff values for each analyzer are indicated by vertical or horizontal dotted lines.

For Anti-HBe results, the overall data are presented in Fig 2A. The Cobas e801 exhibited a high correlation with both the Architect i2000 and Alinity i, second only to the correlation between the Architect i2000 and Alinity i. Because the Atellica assay does not directly report RLU but instead provides converted values positively correlated with Anti-HBe levels, it exhibited an inverse relationship with the other three systems.

A subset of 146 results with values close to the cutoff is presented separately in Fig 2B. Similar to the findings for HBsAg, the Cobas e801 demonstrated strong agreement with the Architect i2000 and Alinity i (second only to the correlation between these two systems) and exhibited a systematic negative bias relative to both. The Atellica IM 1600 classified certain samples, which were determined to be positive by the Architect i2000 or Alinity i, as negative.

### Concordance rates among assay results from the four systems

As expected, the Architect i2000 and Alinity i showed the highest overall concordance rate (Cohen’s κ = 0.953 for HBeAg and 0.971 for anti-HBe). The concordance rates, expressed as Cohen’s κ, were generally low in pairwise comparisons involving the Atellica IM 1600 and other three systems. Notably, the lowest concordance rate for anti-HBe was observed between the Architect i2000 and Cobas e801 (Table 3).

**Table 3:** Inter-Assay Agreement of HBeAg and Anti-HBe Reactivity Across Four Systems.

	Positive agreement	Negative agreement	Cohen’s κ
HBeAg			
Architect vs Alinity	0.932 (0.872–0.965)	1.000 (0.988–1.000)	0.953 (0.920–0.985)
Architect vs Cobas	0.805 (0.724–0.866)	1.000 (0.988–1.000)	0.858 (0.802–0.913)
Architect vs Atellica	0.949 (0.893–0.976)	0.960 (0.932–0.976)	0.892 (0.844–0.939)
Alinity vs Cobas	0.864 (0.787–0.916)	1.000 (0.988–1.000)	0.905 (0.857–0.951)
Alinity vs Atellica	0.982 (0.936–0.995)	0.948 (0.919–0.968)	0.890 (0.841–0.938)
Cobas vs Atellica	0.989 (0.943–0.998)	0.910 (0.875–0.936)	0.807 (0.744–0.870)
Anti-HBe			
Architect vs Alinity	0.996 (0.979–0.999)	0.972 (0.936–0.988)	0.971 (0.948–0.994)
Architect vs Cobas	1.000 (0.985–1.000)	0.665 (0.593–0.730)	0.702 (0.634–0.768)
Architect vs Atellica	1.000 (0.985–1.000)	0.863 (0.801–0.907)	0.886 (0.839–0.931)
Alinity vs Cobas	0.996 (0.979–0.999)	0.674 (0.602–0.739)	0.709 (0.642–0.775)
Alinity vs Atellica	0.992 (0.973–0.998)	0.872 (0.810–0.915)	0.885 (0.838–0.931)
Cobas vs Atellica	0.914 (0.877–0.941)	0.966 (0.915–0.987)	0.831 (0.773–0.888)

Architect, Alinity, Cobas, and Atellica stand for Architect i2000, Alinity i, Cobas e801, and Atellica IM 1600, respectively.

In defining positive or negative agreement, the first assay in a “vs” comparison refers to the reference assay.

Agreements and Cohen’s κ are presented with 95% confidence intervals.

## Discussion

Considering the growing prevalence of high-throughput, fully automated immunoanalyzers in clinical laboratories, harmonization of assay results across different platforms is increasingly important for inter-laboratory quality control. Several previous studies have compared hepatitis B marker results obtained from different assay platforms. In 2006, Chen et al. compared HBV marker results using three automated immunoassay systems: AxSYM and Architect i2000 (Abbott), and the E170 (Roche). Substantial discrepancies were observed among the results from the three platforms, with qualitative agreement being lower for antibody tests than antigen tests. In particular, the differences were notable in the results for anti-HBs and anti-HBe. More recently, a 2018 study conducted by Mixson-Hayden et al. reported that the HBeAg assay on the Vitros platform (Ortho Clinical Diagnostics) showed limited sensitivity and considerable discordance to the results from the ADVIA Centaur system (Siemens). These findings collectively suggest that significant discrepancy would exist among automated assay platforms, which can have important implications for clinical decision-making.

Here, we compared the results of HBeAg and anti-HBe assays among four high-throughput, fully automated analyzers. Although the S/CO or COI is a semi-quantitative value, continued efforts have aimed to derive additional diagnostic value from it. For instance, in anti-HCV testing, the S/CO threshold for each commercial assay has been established to determine positivity with high confidence without a confirmatory test [25,26]. Moreover, previous studies have calculated conversion factors to derive the quantitative unit of HBeAg (PEIU/mL) from S/CO or COI values obtained from commercial automated assays, using the Paul Ehrlich Institute standard [27,28]. Considering this context, we evaluated the quantitative correlation among the index values obtained from each platform (Figs 1 and 2).

Despite sharing the key elements of assay principles, non-negligible differences were observed among the systems. For both HBeAg and Anti-HBe, pairwise comparison among the Architect i2000, Alinity i, and Cobas e801 demonstrated the strongest correlation, whereas the Atellica IM 1600 consistently showed a weaker correlation with each of the other three systems. Since the Architect i2000 and Alinity i, along with their respective assays, originate from the same manufacturer (i.e., Abbott Laboratories) the fundamental principles and core chemistry of the assays are likely similar, if not identical, which explains the strong correlation between the assay index values from these systems.

Although the Cobas e801 showed a strong overall correlation with the Architect i2000 (Fig 1A; R² = 0.9838), its correlation substantially declined in the clinically critical range (Fig 1B; R² = 0.7345). Moreover, in this lower COI range, it exhibited a considerable systematic negative bias relative to the Architect i2000 (slope = 0.5922), resulting in a substantial number of samples that were classified as negative by the Cobas e801 but as positive by the Architect i2000. Therefore, despite the strong quantitative correlation, the Cobas e801 demonstrated notably low positive agreement and only a modest Cohen’s Kappa coefficient (Table 3).

A similar pattern was observed in the anti-HBe assay results, where, as with HBeAg, the Cobas e801 exhibited weaker correlation in the clinically critical low COI range (Fig 2A and B; R² = 0.9487 for overall data, R² = 0.8267 for low-value data only). In this case, a systematic negative bias near the cutoff (slope = 0.6183) led to discrepancies in classification for a considerable number of samples, which were classified as negative by Architect i2000 but as positive by Cobas e801. Consequently, despite the strong quantitative correlation, the Cobas e801 exhibited a notably low negative agreement of 0.665 (Table 3).

To further investigate the underlying causes of these discrepancies, we gathered clinical information on the discrepant samples (Table 4). The 23 samples with discrepant results largely exhibited laboratory findings consistent with effective viral suppression, characterized by inactive HBV DNA replication and normal ALT levels. Except for one case, all patients had undergone prolonged nucleoside analog treatment for 124–3,456 d (median: 1,621.5 d). Therefore, we believe that the non-reactive HBeAg results from the Cobas e801 generally provided a better reflection of the clinical status in these patients.

**Table 4:** Discrepant HBeAg Results Between the Architect i2000 and Cobas e801 with Corresponding Clinical Data.

	Architect i2000				Cobas e801				NA start^a^ (d)	DNA load (IU/mL)	AST (U/L)	ALT (U/L)
Sample No.	HBeAg		Anti-HBe		HBeAg		Anti-HBe					
1	3.01	R	0.35	R	0.69	N	0.27	R	−124	4.08 × 10^1^	33	34
2	2.73	R	1.37	N	0.50	N	0.87	R	−1217	< 10	19	15
3	2.62	R	1.31	N	0.68	N	0.85	R	11	3.51 × 10^3^	34	36
4	2.61	R	1.42	N	0.64	N	0.97	R	−792	<10	20	22
5	2.20	R	1.68	N	0.95	N	1.07	N	−2875	<10	19	23
6	2.05	R	0.08	R	0.43	N	0.03	R	−1521	TND	32	22
7	1.65	R	0.87	R	0.71	N	0.57	R	−3250	TND	30	16
8	1.48	R	1.61	N	0.44	N	1.18	N	−3026	TND	19	19
9	1.42	R	0.27	R	0.45	N	0.19	R	−1722	<10	18	13
10	1.39	R	1.63	N	0.51	N	1.05	N	−2226	<10	30	26
11	1.36	R	1.68	N	0.70	N	1.10	N	−1906	TND	17	10
12	1.32	R	1.62	N	0.77	N	1.08	N	−794	<10	30	30
13	1.30	R	1.30	N	0.85	N	1.04	N	−3002	<10	35	35
14	1.29	R	1.24	N	0.34	N	0.78	R	−817	<10	20	16
15	1.23	R	1.28	N	0.20	N	0.89	R	−305	<10	36	11
16	1.19	R	1.46	N	0.42	N	0.97	R	−1133	TND	21	14
17	1.18	R	1.16	N	0.52	N	0.87	R	−644	TND	23	9
18	1.12	R	1.62	N	0.77	N	0.99	R	−2088	TND	15	13
19	1.08	R	1.51	N	0.43	N	0.98	R	−3459	<10	17	14
20	1.08	R	1.14	N	0.30	N	0.77	R	−979	n/a	25	26
21	1.04	R	1.68	N	0.25	N	1.22	N	−2388	<10	24	24
22	1.04	R	1.66	N	0.80	N	1.19	N	−2536	4.22 × 10^3^	33	29
23	1.01	R	1.58	N	0.33	N	1.01	N	−1061	TND	39	58

ALT, alanine aminotransferase; AST, aspartate aminotransferase; TND, target not detected; N, non-reactive; NA, nucleot(s)ide analog; R, reactive.

^a^Time of NA initiation relative to the laboratory test date (e.g., −124 indicates that NA treatment started 124 d before the test date, whereas 11 indicates that NA treatment started 11 d after the test date).

Another potential application of these data could be the analysis of cases with simultaneous HBeAg/anti-HBe positivity. HBeAg/anti-HBe double positivity has been reported in 1.5–3% of CHB cases, depending on the study [29]. Previous studies suggest an association between HBeAg/anti-HBe double positivity and more pronounced liver damage during active immune clearance, as indicated by elevated ALT levels [29,30]. Although the mechanism underlying double positivity remains to be fully elucidated, findings from cases of HBsAg/anti-HBs double positivity suggest that HBeAg/anti-HBe double positivity may also be attributed to a variant form of HBeAg that escapes *in-vivo* humoral immunity, while binding to anti-HBe *in-vitro* [31]. Notably, the A1896 mutation, which commonly explains the absence of HBeAg in HBeAg-negative CHB, is observed in approximately 40% of HBeAg/anti-HBe double positivity cases [30].

However, in clinical laboratory settings, either HBeAg, anti-HBe, or both are often observed to only slightly exceed the cutoff. Such cases may represent a transient phenomenon wherein a patient is transitioning from the immune active phase to the inactive carrier phase, rather than true double positivity resulting from tentative variant HBV strains. Considering both these possibilities, we examined how the comparator platforms classified samples that exhibited dual positivity on the Architect i2000 (Table 5): samples 1 and 2 showed ALT levels above the upper normal limit, aligning more closely with the immune active phase, whereas samples 3–6 had ALT levels within the normal range, suggesting an interpretation consistent with the inactive carrier phase.

**Table 5:** Samples Identified as Positive for Both HBeAg and Anti-HBe by the Architect i2000.

	Architect i2000				Alinity i				Cobas e801				Atellica IM 1600				NA start^a^ (day)	DNA load (IU/mL)	AST (U/L)	ALT (U/L)
Sample No.	HBeAg		Anti-HBe		HBeAg		Anti-HBe		HBeAg		Anti-HBe		HBeAg		Anti-HBe					
1	7.51	R	0.21	R	7.16	R	0.21	R	12.00	R	0.07	R	52.1	R	7.46	R	−2213	4.60 × 10^2^	54	85
2	3.18	R	0.15	R	2.85	R	0.15	R	2.01	R	0.13	R	11.1	R	3.69	R	0	7.63 × 10^6^	65	46
3	3.01	R	0.35	R	2.48	R	0.34	R	0.69	N	0.27	R	6.44	R	6.11	R	−124	4.08 × 10^1^	33	34
4	2.05	R	0.08	R	1.9	R	0.08	R	0.43	N	0.03	R	1.3	R	8.51	R	−1521	TND	32	22
5	1.65	R	0.87	R	1.32	R	0.81	R	0.71	N	0.57	R	1.37	R	4.67	R	−3253	TND	30	16
6	1.42	R	0.27	R	1.24	R	0.23	R	0.45	N	0.19	R	1.98	R	6.51	R	−1722	< 10	18	18

ALT, alanine aminotransferase; AST, aspartate aminotransferase; TND, target not detected; N, non-reactive; NA, nucleot(s)ide analog; R, reactive.

^a^Time of NA initiation relative to the laboratory test data.

**Fig 3:**
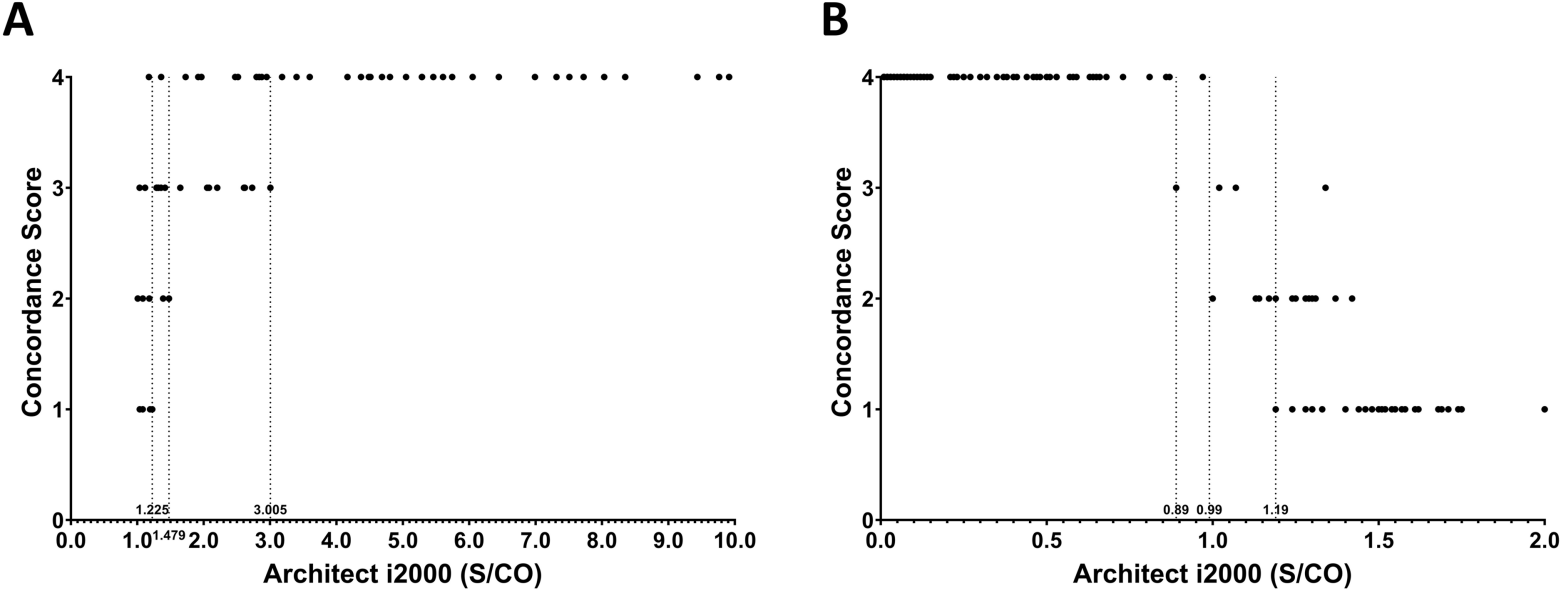
Qualitative concordance of other platforms according to sample-specific S/CO values on the Architect i2000. The y-axis, concordance score, represents the number of platforms that reported the same qualitative result for a given sample (e.g., a score of four indicates that all platforms reported a Reactive result, consistent with the Architect i2000, whereas 1 means that the other three platforms reported a Non-reactive result, differing from the Architect i2000). The cutoff for concordance score is indicated by the dashed line. (A) Results for HBeAg and (B) anti-HBe.

Finally, we explored clinically useful cutoff values that would allow reliable reporting of reactive results when exceeding those thresholds. We examined how each sample was evaluated on the three other analyzers based on the S/CO values measured by the Architect i2000 (Fig 3A and B). For HBeAg, the manufacturer recommends re-examining the initial reactive results (S/CO ≥ 1.0) in duplicate and confirming consistency in at least two of three tests before reporting them as reactive. In our dataset, all samples with S/CO values >3.005 were consistently reported as reactive across all three other analyzers, while those with values above 1.479 were reported as reactive by at least two of the three. Similarly, for anti-HBe, all samples with S/CO values below 0.89 were reported as reactive by all three other analyzers, and those with values below 0.99 were reported as reactive by at least two. These findings suggest that strongly positive cases, such as HBeAg ≥ 3.005 or anti-HBe ≤ 0.89, can be reliably reported as reactive without additional confirmatory testing.

This study has several limitations. First, the sample size was relatively small. The number of samples meeting specific conditions (e.g., HBeAg/anti-HBe double positivity) was insufficient to draw definitive conclusions. Moreover, our dataset did not include additional clinical data related to the infection status of patients with CHB, such as the age, sex, presence or absence of cirrhosis, and extent of fibrosis estimated by liver stiffness testing or liver imaging. Similarly, viral factors such as HBV genotype and information on coinfection status with other hepatitis viruses or HIV were unavailable. In addition, a definitive reference method for HBeAg testing was lacking, making it impossible to determine a conclusive standard in cases of platform discrepancies. For such discrepant cases, the true serological status could only be inferred based on HBV DNA loads and laboratory markers of liver injury (e.g., AST, ALT). Finally, we did not follow the confirmatory retesting algorithms recommended by the manufacturers for testing and interpretation. Therefore, the actual performance of each platform, as well as concordance between platforms, may be higher than what was estimated in our study.

In conclusion, this study evaluated the performance of four fully automated immunoanalyzers and compared their results. Notably, we observed significant inter-platform differences in the critical range near the cutoff, which can have important implications in clinical decision-making. Future studies are needed to further investigate these discrepancies and contribute to harmonize assay results across platforms.
